# P-2348. Predictors of Dengue Outcome after Adoption of a Management Protocol

**DOI:** 10.1093/ofid/ofae631.2500

**Published:** 2025-01-29

**Authors:** Keshav Raj Sigdel, Rehan Pradhan, Saugat Lamichhane, Nora Ranjitkar, Janak Koirala

**Affiliations:** Patan Academy of Health Sciences, Lalitpur, Bagmati, Nepal; Patan Academy of Health Sciences, lalitpur, Bagmati, Nepal; Patan Academy of Health Sciences, Lalitpur, Bagmati, Nepal; Patan Academy of Health Sciences, Lilitpur, Bagmati, Nepal; Patan Academy of Health Sciences, Lalitpur, Bagmati, Nepal

## Abstract

**Background:**

Dengue caused a large outbreak in Nepal with over 54,000 reported cases in 2022. Once considered a disease of tropics, dengue has expanded to subtropical and temperate regions of Nepal including Kathmandu valley. A standard protocol, which included screening criteria for admission and treatment based on severity, was adopted at our hospital which is located at the epicenter of 2022 outbreak.

**Methods:**

We enrolled 261 patients admitted to Patan Academy of Health Sciences between August and October, 2022. We analyzed patients’ baseline clinical and laboratory characteristics as predictors of outcomes including duration of stay and mortality.

**Results:**

The mean age of the patients was 40.9 (±19.1) years with female predominance (54.4%). Most patients were from Lalitpur district followed by Kathmandu, which are the main catchment areas of the hospital and epicenter of 2022 outbreak. Most common clinical presentations included fever (93.5%),followed by vomiting (65.5%), myalgia (52.1%), headache (45.6%), mucosal bleeding (18.4%), eye pain (16%), anorexia (13.8%), loose stools (13.8%), and rash (10%). The patient’s age correlated directly with both systolic and diastolic blood pressures (p=0.002 and 0.01, respectively), and inversely with platelet counts (p=0.002).

Duration of hospital stay (median 3, IQR 2 days) correlated with aspartate aminotransferase (median 141 U/L) and alanine aminotransferase (median 90.5 U/L) levels at presentation (p< 0.001 and p=0.03, respectively). Hemorrhagic rash occurred in 10 patients and was associated with low platelets at day 1 of admission (p=0.01). There were no deaths.

**Conclusion:**

Elevated aminotransferases as a marker of hepatocellular injury was the main predictor of longer duration of hospital admission. With adoption of a standard screening and treatment protocol, despite varied clinical presentations and complications, no fatalities occurred among the 261 adult patients in our hospital. This underscores the importance of early detection and proper management in effectively combating the disease and preventing mortality.

**Disclosures:**

All Authors: No reported disclosures

